# DOT or SAT for Rifampicin-resistant tuberculosis? A non-randomized comparison in a high HIV-prevalence setting

**DOI:** 10.1371/journal.pone.0178054

**Published:** 2017-05-18

**Authors:** Erika Mohr, Johnny Daniels, Busisiwe Beko, Petros Isaakidis, Vivian Cox, Sarah Jane Steele, Odelia Muller, Leigh Snyman, Virginia De Azevedo, Amir Shroufi, Laura Trivino Duran, Jennifer Hughes

**Affiliations:** 1 Médecins Sans Frontières (MSF), Khayelitsha, South Africa; 2 Médecins Sans Frontières (MSF), South African Medical Unit, Cape Town, South Africa; 3 University of Cape Town (UCT), Center for Infectious Disease Epidemiology and Research, School of Public Health and Family Medicine, Cape Town, South Africa; 4 Médecins Sans Frontières (MSF), Cape Town, South Africa; 5 City of Cape Town Health Department, Cape Town, South Africa; McGill University, CANADA

## Abstract

**Background:**

Daily directly-observed therapy (DOT) is recommended for rifampicin-resistant tuberculosis (RR-TB) patients throughout treatment. We assessed the impact of self-administered treatment (SAT) in a South African township with high rates of RR-TB and HIV.

**Methods:**

Community-supported SAT for patients who completed the intensive phase was piloted in five primary care clinics in Khayelitsha. We compared final treatment outcomes among RR-TB patients initiating treatment before (standard-of-care (SOC)-cohort, January 2010-July 2013) and after the implementation of the pilot (SAT-cohort, January 2012-December 2014). All patients with outcomes before January 1, 2017 were considered in the analysis of outcomes.

**Results:**

One-hundred-eighteen patients in the SOC-cohort and 174 patients in the SAT-cohort had final RR-TB treatment outcomes; 70% and 73% were HIV-co-infected, respectively. The proportion of patients with a final outcome of loss to follow-up (LTFU) did not differ whether treated in the SOC (25/118, 21.2%) or SAT-cohort (31/174, 17.8%) (*P* = 0.47). There were no significant differences in the time to 24-month LTFU among HIV-infected and uninfected patients (HR 0.90, 95% CI: 0.51–1.6, *P* = 0.71), or among patients enrolled in the SOC-cohort versus the SAT-cohort (HR 0.83, 95% CI: 0.49–1.4, *P* = 0.50) who received at least 6-months of RR-TB treatment.

**Conclusion:**

The introduction of SAT during the continuation phase of RR-TB treatment does not adversely affect final RR-TB treatment outcomes in a high TB and HIV-burden setting. This differentiated, patient-centred model of care could be considered in RR-TB programmes to decrease the burden of DOT on patients and health facilities.

## Introduction

Universally rifampicin-resistant tuberculosis (RR-TB) treatment outcomes remain poor, with treatment success rates of approximately 50%[1]. Rates of loss to follow-up (LTFU), treatment failure and death remain high due to toxic, relatively ineffective treatment options[2]^,^ [3]^,^ [4]. Treatment lasts up to 24-months and requires patients to administer treatment daily under directly observed therapy (DOT)[4].

Research suggests DOT is not the solution to poor adherence[5]^,^[6]^,^[7]; patients perceive that facility based DOT perpetuates stigma, hinders collection and administration of treatment, and inhibits return to daily activities[8][9]. Furthermore DOT imposes restraints on clinical resources and time for clinicians to effectively manage patients[8]. Studies investigating dual adherence in HIV-infected RR-TB patients found adherence to anti-retroviral therapy (ART) was significantly higher at 6-months when compared to RR-TB treatment[10]; co-infected patients reported greater tolerability to and less perceived stigma associated with ART[11]. Supervised RR-TB treatment administration has led to negative experiences between patients and RR-TB service providers, potentially negatively impacting retention-in-care (RIC) [10]^,^[11].

The transition from the intensive phase-6-8 months of treatment including an injectable- to the continuation phase is a high-risk period for LTFU[12], after which 2/3 of patients who experience LTFU are lost due to clinical improvement[13]. Factors associated with LTFU include gender, age, previous TB, substance abuse, and distance from the clinic[13]^,^[14]^,^[15]. An outcome of LTFU has implications for RR-TB control strategies; patients LTFU potentially contribute to ongoing community transmission of RR-TB and are more likely to experience recurrent RR-TB than those successfully treated [16]. Patients LTFU additionally have increased risk of mortality following treatment cessation[2]^,^ [17]. Strategies to reduce LTFU include the provision of patient support[8], education, smaller treatment cohorts[18], outpatient DOT[9], and community health workers to provide treatment[18].

In South Africa, where high rates of RR-TB are driven by the HIV epidemic[19], the proportion of patients LTFU range from 20%-31%[14]^,^[20]^,^[21]. In 2007 Médecins Sans Frontières (MSF), in collaboration with local partners, implemented a patient-centred model of decentralised care for RR-TB patients in Khayelitsha, South Africa [21]^,^[22]^,^[23]. This model entailed the provision of RR-TB treatment and care at the primary health care level. The programme has led to increased case detection and treatment initiation rates, and reduced time to RR-TB treatment initiation[21],[23],[24]. However, LTFU rates remain at approximately 30%[21] thus a variety of adherence-support strategies have been implemented[25]^,^ [26].

Primary care clinics often provide a supply of RR-TB medication for self-administration despite current treatment guidelines recommending DOT (standard-of-care, SOC)[27]. MSF and local partners piloted a programme to formalize and strengthen community-supported self-administered treatment (SAT) for RR-TB patients. The overall aim of the SAT pilot programme was to demonstrate that there is no change in rates of LTFU when selected patients are given a supply of RR-TB medication to self-administer at home. The objective of this analysis was to describe final treatment outcomes, specifically LTFU rates, among patients in Khayelitsha before and after the SAT programme implementation.

## Materials and methods

### Setting

Khayelitsha is a peri-urban township outside of Cape Town, South Africa with a population of approximately 450,000 people, most of whom reside in informal settlements[28]. Approximately 200 patients are diagnosed with RR-TB annually with a case notification rate of 55/100,000[21]; HIV co-infection rates are 70%[29]. The standard RR-TB treatment regimen provided to patients contained all or most of the following drugs: kanamycin, moxifloxacin, pyrazinamide, ethambutol, terizidone, ethionamide and high dose isoniazid. This regimen did not differ for patients in the SAT versus SOC cohorts.

### Standard of care: Directly observed therapy

All patients treated for RR-TB in Khayelitsha, South Africa are to receive DOT as per the national treatment guidelines [27]. This treatment model requires patients to attend the clinic five days per week, where treatment is administered with the support of clinical medical staff. DOT is to be provided for the entire 24-months of treatment. All patients receiving DOT receive four standardized counseling sessions provided by trained RR-TB counselors upon diagnosis, treatment initiation, during the intensive phase of treatment and upon the completion of the intensive phase of treatment. Once monthly these patients were assessed clinically by a medical officer and laboratory parameters were tested in order to monitor treatment response. Attempts to trace patients LTFU are made telephonically or via home visits by counselors or local community care workers (CCWs) upon treatment interruption; patients not linked back to care are assigned an outcome of LTFU after two months of consecutive treatment interruption and unsuccessful tracing efforts.

For the sake of this analysis patients who were treated in clinics where DOT was the recommended model of care, were considered to be enrolled in the SOC-cohort.

### Self-administered treatment

Following completion of the intensive phase of treatment, a RR-TB counselor conducted a tailored counseling session with the patient to congratulate and encourage the patient to continue treatment and discuss the option of receiving SAT. Patients were assessed for SAT based on: treatment adherence record (for RR-TB and concomitant diseases), clinical status and adverse events requiring ongoing monitoring. Local CCWs were assigned to potential SAT patients; prior to SAT enrollment the CCW conducted a home visit to assess the social situation, identify a treatment supporter, and determine adherence barriers. Weekly meetings in each clinic were attended by CCWs, doctors, RR-TB professional nurses and MSF counselors to discuss patients for SAT and those approved were enrolled in SAT for the remainder of treatment if they provided verbal consent. Patients were enrolled if they were no longer receiving an injectable agent, gave verbal consent and met the above stated assessment criteria. At initial implementation in each clinic SAT was offered immediately to eligible patients already in the continuation phase, regardless of injectable completion date.

Once patients were enrolled in the SAT pilot programme they received an adherence counseling session by a dedicated MSF counselor, where medications were reviewed, a pillbox was issued and adherence barriers were addressed. Enrolled patients received a weekly or monthly supply of RR-TB medications, depending on clinic and patient preference. CCWs visited SAT patients weekly initially, and monthly once patients were deemed to be doing well in the programme, for the duration of treatment to offer support and identify adherence challenges. These processes were unique to the patients enrolled in the SAT pilot programme; all other patients received DOT and were required to go to the clinic daily to collect and take treatment.

**Fig 1:**
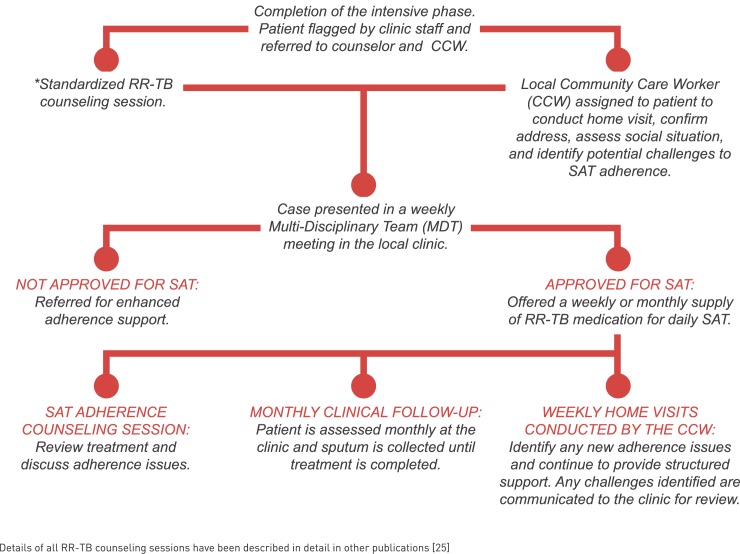
Outline of activities included in the SAT pilot programme.

Patients found to be interrupting treatment (>2 weeks) were referred for review by a doctor and enhanced adherence counseling by the RR-TB counselor, and if necessary, were returned to clinic DOT. If LTFU, these patients are traced according to the SOC, thus there were no differences in the ascertainment of LTFU based on the RR-TB treatment model (SOC versus SAT). All SAT patients attended their clinic monthly for clinical assessment and routine monitoring tests which were also conducted monthly for those receiving DOT (**Fig 1**).

SAT implementation was progressive across 5/10 primary care clinics in Khayelitsha from 2012 to 2015; initial pilot clinics were chosen based on available resources, functionality, and willingness of staff to participate.

### Cohort selection

Patients were considered for the SOC-cohort if they initiated treatment at least 6-months prior to SAT implementation in their specific clinic, in order to limit the number of patients in the SOC-cohort who received SAT. Treatment initiation times for inclusion in the SOC-cohort ranged from January 2010-July 2013. To avoid bias, patients were considered for the SAT-cohort if they started treatment in their clinic at least 6-months after SAT was implemented so that patients who were enrolled in SAT near the end of their treatment course were not included in the cohort. Treatment initiation times for inclusion in the SAT-cohort ranged from January 2012-December 2014.Patients were excluded from the SOC and SAT-cohorts if they had an outcome within 6-months of RR-TB treatment initiation (had an outcome before the end of the intensive phase of treatment). Patients in the SOC and SAT-cohorts were included in the analysis of final outcomes if they had a final treatment outcome before January 1, 2017.

### Definitions

Pulmonary tuberculosis (PTB) and extra pulmonary tuberculosis (EPTB) were defined as *Mycobacterium tuberculosis* (*M*. *tuberculosis*) which affects the lungs and other sites, respectively; patients with both PTB and EPTB were defined as *M*. *tuberculosis* which affects both the lungs and other sites[30]. RR-TB treatment outcomes including treatment success (treatment cure or completion), LTFU and death were defined in line with WHO recommended definitions; LTFU was defined as a patient whose treatment was interrupted for two consecutive months or more[4],[30]. Treatment failure was defined according to South African National Drug Resistant TB guidelines as failure to culture convert after 6–8 months of RR-TB treatment or 6–8 months of consecutive positive cultures[27]. The intensive phase was defined as the first 6-months of RR-TB treatment. Standard and modified regimens were provided as per National Department of Health SOC for RR-TB treatment[27]. Baseline CD4 count was defined as the CD4 count taken within 2 months of RR-TB diagnosis.

### Outcome of interest

The outcome of interest in this study was a final RR-TB treatment outcome of LTFU; we aimed to determine if there were differences in LTFU among those treated in the SOC and SAT-cohorts.

### Data collection & analysis

Routine programmatic RR-TB data collected for the decentralised RR-TB programme (retrospective) were linked with data collected on paper registers for this pilot (prospective). Data collected for the decentralised programme included date of RR-TB diagnosis, date of RR-TB treatment initiation, sex, age at RR-TB diagnosis, TB treatment history, RR-TB disease classification, RR-TB regimen, RR-TB disease site, HIV status, CD4 count at RR-TB diagnosis, anti-retroviral (ART) initiation date, final RR-TB outcome, and date of RR-TB treatment outcome. These data were collected from the national electronic RR-TB register, paper registers, and clinical files. Data collected specifically for the SAT programme included date of MDT presentation, outcome of MDT presentation (placed out without a CCW, placed out with a CCW, not placed out), and reversion to clinic DOT. These data were collected by MSF counselors involved in the identification of patients for presentation to the MDT committee for SAT enrollment.

STATA/IC version 14.1 was used for this analysis; differences in clinical and demographic characteristics and final treatment outcomes assigned before January 1, 2017, stratified by patients initiating RR-TB treatment in the SOC and SAT- cohorts were described. A sub-analysis investigated final outcomes among patients who received SAT only. Chi-squared and Fisher’s exact tests were used for statistical comparison of categorical variables, while Wilcoxon rank-sum tests were used for continuous variables; P-values <0.05 were considered statistically significant. Kaplan Meier (KM) Curves were used to investigate differences in time to 24-month LTFU (censor: death, treatment failure, transfer out or treatment completion) among patients who completed at least 6-months of treatment in the SOC and SAT-cohorts and among those HIV-infected and uninfected.

### Ethics

The analysis of this programme was covered by pre-existing ethical approval for the ‘Evaluation of a decentralised programme for rifampicin-resistant tuberculosis care and treatment in Khayelitsha’ (HREC 540–2010) granted by the University of Cape Town Human Research Ethics Committee. Informed consent was not required by the ethics committee for this study as it involved a retrospective analysis of routinely collected, programmatic data. This study was conducted in line with the STROBE checklist for reporting on observational cohorts (http://www.strobe-statement.org/).

## Results

Among patients initiated on treatment in five Khayelitsha clinics, 160 (70% HIV infected) and 244 (74% HIV infected) were included in the SOC and SAT-cohorts, respectively. TB treatment history was the only statistically different variable between cohorts (p<0.01) (**Table 1**).

**Table 1:** Clinical and demographic characteristics of patients initiated on RR-TB treatment in the SOC-cohort (January 2010—July 2013) versus the SAT-cohort (January 2012—December 2014).

Patients Started RR-TB Treatment	SOC-cohort	SAT-cohort	*P*-value
	N = 160	N = 244	
	n (%)	n (%)	
**Sex**			
Male	94 (58.8)	129 (52.9)	
Female	66 (41.2)	115 (47.1)	0.25
Median Age at diagnosis (years) (IQR)	33.9 (27.2–41.9)	32.7 (27.2–40.6)	0.38
**TB Treatment History**			
No TB treatment history	38 (23.7)	98 (40.2)	
Previous treatment with 1^st^ line drugs	103 (64.4)	113 (46.3)	
Previous treatment with 2^nd^ line drugs	19 (11.9)	33 (13.5)	<0.01
**RR-TB Regimen**			
Standard regimen*	142 (88.7)	217 (88.9)	
Second-line resistance (modified regimen)	18 (11.3)	27 (11.1)	0.95
**Disease Site**			
PTB	149 (93.1)	220 (90.2)	
EPTB only	8 (5.0)	22 (9.0)	
Unknown	3 (1.9)	2 (0.8)	0.22
HIV-infected	112 (70.0)	180 (73.8)	0.41
Median Baseline CD4 count cells/mm^3^ among HIV-infected (IQR)	108 (46.5–241.5)	159 (47–346)**	0.28
On ART at RR-TB diagnosis	45/112 (40.2)	81/180 (45.0)	0.42
Median months on ART (IQR)	10.5 (1.8–27.2)	12.0 (2.1–43.2)	0.26

*Standard Regimen = pyrazinamide/ethionamide/high dose isoniazid/kanamycin/moxifloxacin/

ethambutol/terizidone

**9 patients missing baseline CD4 count

RR-TB, rifampicin-resistant tuberculosis; SOC, standard of care; SAT, self-administered treatment; IQR, interquartile range; TB, tuberculosis; PTB, pulmonary tuberculosis; EPTB, extra-pulmonary tuberculosis; ART, anti-retroviral therapy.

**Fig 2:**
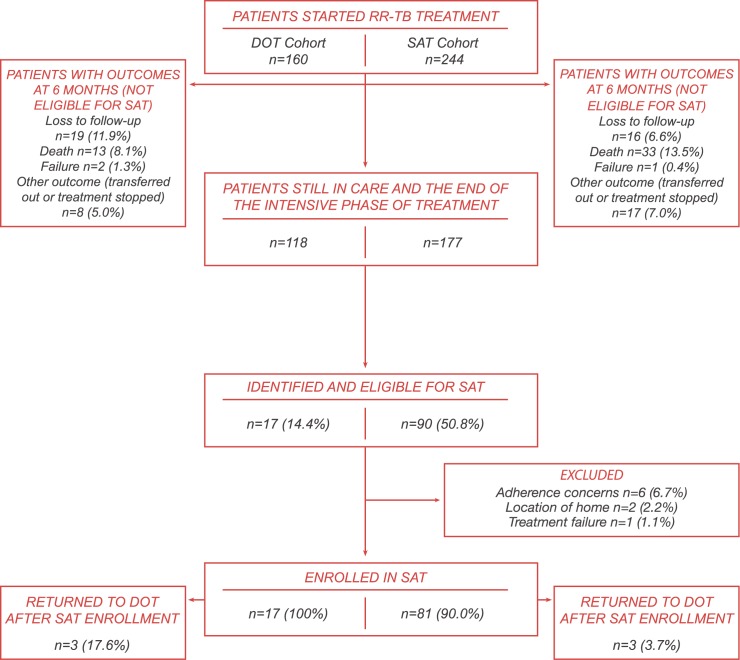
Patient eligibility for SAT among patients initiating RR-TB treatment from January 2010 through July 2013 in the SOC-cohort and January 2012 through December 2014 in the SAT-cohort.

Forty two (26.3%) and 67 (27.4%) patients initiating treatment in the SOC and SAT-cohorts had a treatment outcome before 6-months and were excluded from further analysis (**Fig 2**).Most of the remaining 118 patients in the SOC-cohort continued treatment after 6-months as per SOC, however 17 (14.4%) were later considered for and received SAT, but only for a short period as median time to SAT-enrollment was 14.8-months (IQR 12.8–20.3). Ninety (50.8%) of the 177 patients in the SAT-cohort were considered for SAT due to the phased implementation of the pilot; of these, 81 (90.0%) were enrolled. Reasons for non-enrollment included adherence concerns (n = 6), location of home (n = 2), or treatment failure (n = 1). Median time to SAT enrollment was 7.8-months (IQR 6.4–9.6) following RR-TB treatment initiation.

**Fig 3:**
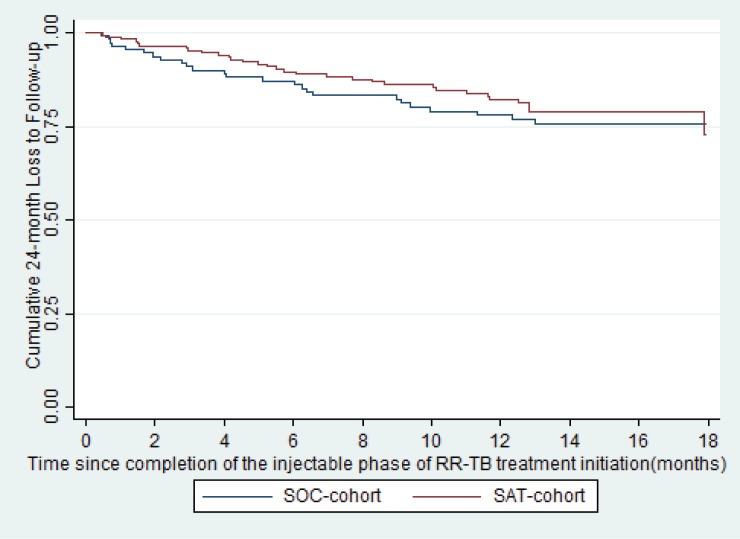
Loss to follow-up by 24-months among patients who completed at least 6-monts of treatment in the SOC versus SAT-cohorts (p = 0.50).

Final treatment outcomes were available for 118 (70.3% HIV-infected) and 174 (72.9% HIV-infected) patients in the SOC and SAT-cohorts, respectively (**Table 2**) by January 1, 2017. There were no significant differences in final treatment outcomes among patients who received at least 6-months of treatment in the SOC and SAT-cohorts (**Table 2**); 21.2% (25/118) and 17.8% (31/174) had final outcomes of LTFU in the SOC and SAT-cohorts respectively (*P* = 0.47). The proportion of patients LTFU did not significantly differ based on HIV status (39/210 [18.6%] for HIV-infected versus 17/82 [20.7%] for HIV-uninfected, P = 0.67). Additionally, time to LTFU at 24-months did not significantly differ among patients who received at least 6-months of treatment in the SOC and SAT-cohorts (Hazard Ratio (HR) 0.83, 95% Confidence Interval (CI): 0.49–1.4, *P* = 0.50, **Fig 3**), nor did it significantly differ based on HIV status (HR 0.90, 95% CI: 0.51–1.6, *P* = 0.71).

**Table 2:** Final treatment outcomes assigned before January 1, 2017 for patients enrolled in the SOC (January 2010—July 2013) and SAT (January 2012—December 2014) cohorts who completed at least 6 months of treatment.

Final RR-TB treatment outcomes	SOC-cohort n = 118 n (%)	SAT-cohort n = 174 n (%)	*P*-value
**Treatment success**	66 (55.9%)	99 (56.9%)	0.87
**Loss from Treatment**	25 (21.2%)	31 (17.8%)	0.47
**Death**	6 (5.1%)	15 (8.6%)	0.25
**Treatment Failure**	5 (4.2%)	7 (4.0%)	0.93
**Not Evaluated**	16 (13.6%)	22 (12.6%)	0.82

RR-TB, rifampicin-resistant tuberculosis; SOC, standard of care; SAT, self-administered treatment.

## Discussion

Our findings indicate that SAT is a potential feasible alternative to clinic DOT for long term RR-TB treatment as there was no difference in the proportion of patients LTFU among patients initiating treatment before or after the implementation of the SAT programme. These results suggest that introduction of SAT, where patients are encouraged to take responsibility for their own treatment adherence, does not lead to more treatment interruption or a reduction in the proportion of patients retained in care at 24-months. SAT poses fewer challenges to both patients and healthcare providers than DOT (i.e. daily transportation to the clinic and daily burden on clinic resources), and offers more social benefits and improved quality-of-life (QOL) for patients[6]^,^[8]. Our findings may support the implementation of community-supported SAT into RR-TB treatment programmes, potentially assisting in the reduction of the burden posed by DOT on patients and clinics.

The introduction of rapid diagnostics (i.e. Xpert MTB/RIF) in 2011 allowed for screening of all TB suspects, rather than just high risk groups such as previously treated TB patients[23]^,^ [31], which likely led to the lower proportion of patients with a TB treatment history in the SAT-cohort. RR-TB patients with a history of second-line TB treatment were more likely to be LTFU at 24-months; such patients are more like to have treatment fatigue and a history of LTFU[7]^,^[13]. The high HIV-infection rates were not significantly different between the cohorts and the overall rate of LTFU did not differ based on HIV status. The large proportion of co-infected patients not on ART at RR-TB diagnosis in both cohorts was concerning. This suggests patients are presenting with low CD4 counts, and only diagnosed with HIV or brought back into care after loss from ART at RR-TB diagnosis. Case detection efforts should be intensified given universal access to ART in South Africa.

Evidence suggests that DOT introduces barriers to optimal treatment adherence[7]^,^[8]^,^ [32]. SAT opponents argue that placing responsibility for treatment adherence on the patient will lead to increased LTFU[18] however, our findings do not reflect this; in fact, there appears to be a lower LTFU rate among patients in the SAT cohort however this difference was not statistically significant. RR-TB treatment programmes should consider patient convenience and QOL while actively promoting RIC; lessons learnt from HIV programmes could be applied to improve RIC[11].

Studies investigating adherence in drug-sensitive TB patients show that patients required to attend the clinic daily were twice as likely to miss treatment doses than those requiring fewer clinical visits[6]. Additionally, DOT did not significantly improve treatment success when compared to SAT[5]; community-based adherence support was a feasible alternative to DOT[33]. A systematic review and meta-analysis of MDR-TB outcomes among patients on DOT for a full treatment course versus intensive phase DOT found there was no difference in outcomes[34]. We also found no difference in LTFU at 24-months whether or not treatment was continued under clinic DOT; our findings suggest that implementation of community-supported SAT does not lead to increased LTFU and therefore might be a more sustainable model of care given its reduced burden on the health care system.

### Limitations

This study had several limitations, however, the study might have been too small to see an effect of these limitations. As this was a non-randomized comparison, there are threats to the validity of the results presented. As the criteria to receive SAT were subjective and patients who were doing well on or adherent to RR-TB treatment were selected for the intervention, there might have been an overestimation of the effect of SAT. This was potentially minimized however, by conducting an intent-to-treat analysis. Due to the slow and phased implementation and initial reluctance by care providers to endorse the pilot, some eligible patients in pilot clinics were never offered SAT; limited resources to prepare and support large numbers of patients initiating SAT at once made this impossible. The eligibility criteria for enrollment in SAT were very subjective, based on adherence and clinical response to treatment, resulting in selection bias for enrollment into SAT. Time on SAT was a potential confounder in this analysis as some patients only enrolled onto SAT much later in treatment. However, median time to SAT enrollment was 7.8 months, indicating that patients completing the intensive phase were prioritized over those nearing continuation phase treatment completion. Thus the small number of patients who received SAT later in treatment likely had little effect on the results. Additionally, patients in the SOC-cohort might have received an informal version of SAT as facilities occasionally provided a supply of medications for self-administration to relieve pressure on the clinic, despite clinic DOT being the SOC. This contamination may have resulted in an underestimation of the true effect of SAT. These patients however, did not receive the specialized counseling and ongoing community support integral to the SAT pilot programme. Additionally, due to phased implementation of the pilot some patients in the SOC-cohort actually were offered SAT through the programme. This study was based on operational data and has limited statistical power but provides sufficient evidence to encourage future research of this intervention in larger studies. Despite these limitations, this was a study of routine, programmatic data which provided an overview of final RR-TB treatment outcomes among patients who received SAT in pilot clinics in a programmatic setting. Inclusion of all patients still on treatment at the end of the intensive phase in both cohorts limited any overestimation of the impact of SAT. Further research, including qualitative studies, is planned to determine patients’ and care providers’ perspectives regarding SAT, the impact of SAT implementation on healthcare providers and facilities and the utility of SAT among less adherent patients.

## Conclusion

Twenty four-month LTFU rates for RR-TB patients did not differ between the SOC versus SAT-cohorts, suggesting that introduction of SAT does not negatively impact RIC in a programmatic setting with high RR-TB and HIV burdens. Community-based SAT is a patient-centred model of treatment and care delivery designed to address the reality of overwhelmed patients and overburdened clinics, with structured patient-support components. Given the potential benefits of SAT for patients and healthcare systems, it might pose as a differentiated model of care in settings similar to Khayelitsha with high burdens of RR-TB. Further studies are needed in order to confirm these findings and determine the utility of SAT in other programmatic settings. SAT alone is not the solution to improving the patients treatment journey; RR-TB programmes need comprehensive strategies, including specialized interventions focusing on patients at high risk for LTFU to address the unchanged LTFU rates. There is an urgent need to focus on the complex psycho-social needs of the patient to ensure treatment completion; lessons learned from HIV programmes could greatly help RR-TB programmes in their transition to patient-centred models of care[8]^,^[10]. Additionally, shorter, less toxic, injectable free, and more efficient treatment regimens with new and improved RR-TB drugs are imperative for improving patient adherence[9], treatment success and decreasing high mortality among RR-TB patients[35]^,^ [36]^,^[37].

## Supporting information

S1 File STROBE statement checklist for observational cohort studies. (DOC)

S2 File Database for the study “DOT or SAT for rifampicin-resistant tuberculosis? A non-randomized comparison in a high HIV-prevalence setting”. (XLS)
